# Development of a Novel Zebrafish Model for Type 2 Diabetes Mellitus

**DOI:** 10.1038/s41598-017-01432-w

**Published:** 2017-05-03

**Authors:** Liqing Zang, Yasuhito Shimada, Norihiro Nishimura

**Affiliations:** 1 Graduate School of Regional Innovation Studies, Mie University, Tsu, Mie Japan; 2 Department of Integrative Pharmacology, Mie University Graduate School of Medicine, Tsu, Mie Japan; 3 Department of Bioinformatics, Mie University Life Science Research Center, Tsu, Mie Japan

## Abstract

Obesity is a major cause of type 2 diabetes mellitus (T2DM) in mammals. We have previously established a zebrafish model of diet-induced obesity (DIO zebrafish) by overfeeding *Artemia*. Here we created DIO zebrafish using a different method to induce T2DM. Zebrafish were overfed a commercially available fish food using an automated feeding system. We monitored the fasting blood glucose levels in the normal-fed group (one feed/day) and overfed group (six feeds/day) over an 8-week period. The fasting blood glucose level was significantly increased in DIO zebrafish compared with that of normal-fed zebrafish. Intraperitoneal and oral glucose tolerance tests showed impaired glucose tolerance by overfeeding. Insulin production, which was determined indirectly by measuring the EGFP signal strength in overfed *Tg(−1.0ins:EGFP)* ^*sc1*^ zebrafish, was increased in DIO zebrafish. The anti-diabetic drugs metformin and glimepiride ameliorated hyperglycaemia in the overfed group, suggesting that this zebrafish can be used as a model of human T2DM. Finally, we conducted RNA deep sequencing and found that the gene expression profiling of liver-pancreas revealed pathways common to human T2DM. In summary, we developed a zebrafish model of T2DM that shows promise as a platform for mechanistic and therapeutic studies of diet-induced glucose intolerance and insulin resistance.

## Introduction

The International Diabetes Federation reported that in 2013, more than 387 million people were living with diabetes and more than 90% of all diabetes cases were examples of type 2 diabetes mellitus (T2DM)^1^. T2DM is characterized by insufficient secretion of insulin from the β-cells of pancreatic islets, coupled with impaired insulin action at target tissues such as muscle, liver and adipose tissue (a condition termed insulin resistance)^2^. Obesity is a major independent risk factor for developing T2DM, and more than 90% of people with T2DM are overweight or obese. Intra-abdominal adipocytes release a large amount of non-esterified fatty acids into the circulation. Increased flux of these fatty acids to the liver and muscle promotes lipotoxicity and altered insulin action, leading to insulin resistance and deterioration of glucose homeostasis^3^. People with insulin resistance need more insulin to help glucose enter the cells. To compensate, the pancreas tries to keep up with the increased demand for insulin, but eventually becomes damaged and fails to produce the required amount.

Progress in the development of anti-diabetic treatments is improving the prognosis of T2DM. However, patients with diabetes should continue to monitor their blood glucose and diabetes medications throughout their lives to prevent worsening of the disease and diabetic complications. Since 1981, 37 anti-diabetic drugs have been approved by the Food and Drug Administration (FDA) for their ability to increase insulin secretion, insulin sensitivity, and/or decrease the rate of glucose absorption from the gastrointestinal tract^4^. Important drug targets have been identified that play a central role in T2DM therapy. For instance, thiazolidinediones (TZDs) bind to and activate PPARγ to improve insulin sensitivity^5^; and biguanides and TZDs act by directly or indirectly activating AMPK^6, 7^. These drugs are effective for the prevention of hyperglycaemia and diabetic complications such as cardiovascular disorders; however, they cannot repair pancreatic damage. The mechanisms of insulin resistance and glucotoxicity in pancreas need to be elucidated so that new drug targets can be identified and new anti-diabetics developed.

Animal models of abnormal glucose metabolism are undoubtedly useful in this regard with their offer of new insights into T2DM. Numerous animal models of T2DM have been developed using: 1) spontaneous or planned genetic derivation^8, 9^; 2) dietary/nutritional induction^10^; 3) chemical induction^11^; 4) surgical manipulation^12^; 5) transgenic/knock-out manipulation^13^; or 6) a combination of the above^14^. Most of the available models are rodent-based, which have drawbacks in that they are labour intensive and because of ethical issues, only small groups of animals can be used. To overcome these limitations, the zebrafish (*Danio rerio*) has been increasingly used to study diabetes and its related diseases, chosen because of the high similarities in organ physiology and metabolism between zebrafish and mammals. Recent studies have identified the zebrafish as an excellent system for the discovery and characterization of new diagnostic and therapeutic targets for metabolic diseases, including visceral adiposity^15, 16^, non-alcoholic steatohepatitis^17^, atherosclerosis^18^ and diabetes^19^. Several zebrafish models of diabetes have been established using toxin-mediated ablation of β-cells^20, 21^, ENU-induced mutagenesis screening^22^, and morpholino and gRNA/Cas9 mediated knockdown techniques^23^. Of these, a strain of zebrafish with a mutation in pancreatic and duodenal homeobox 1 (*pdx1*, also known as insulin promoter factor 1, *ipf1*), which is a gene linked to a genetic cause of T2DM in humans^24^, is presented as a genetic model of T2DM^22^. However, a null mutation in *pdx1* reduced the fish’s body size and decreased their viability, limiting the application of this strain to studies of T2DM.

We have previously established a zebrafish model of diet-induced obesity (DIO) by overfeeding with *Artemia* ^25^. In this study, we modified our overfeeding method by changing the fish food and using an automatic feeder. We found that these new DIO zebrafish show higher blood glucose after a period of fasting (FBG) than normally-fed zebrafish. We further demonstrate this hyperglycaemic zebrafish to be a useful model for T2DM through glucose tolerance testing and measurements of insulin production and glycaemic response to human anti-diabetic drugs. In addition, a RNA-seq analysis of liver-pancreas tissues reveals that the T2DM zebrafish shares pathological pathways with humans.

## Materials and Methods

### Animals, diets and experimental design

All animal procedures were approved by the Ethics Committee of Mie University, were performed according to the Japanese animal welfare regulation ‘Act on Welfare and Management of Animals’ (Ministry of Environment of Japan) and complied with international guidelines. Zebrafish (*AB* and *Tg(−1.0ins:EGFP)* ^*sc1*^ strain (referred to as ins-EGFP); the Zebrafish International Research Centre, Eugene, OR, USA) were maintained in our facility according to established protocols^26^. Male healthy adult zebrafish (4–6 months old) were assigned to either an overfeeding or a control group with 5 fish per 2 L tank. DIO zebrafish were fed 120 mg per fish per day of a commercially available fish food (Otohime B2; Marubeni Nisshin Feed, Tokyo, Japan) divided over six daily feedings using an automated feeding system (Marukan, Osaka, Japan). Non-DIO zebrafish were fed 20 mg per fish per day of Otohime B2 once daily. Otohime B2 contains a minimum of 11% crude fat, 51% crude protein, 2.3% crude calcium, 1.5% phosphorous, a maximum of 15% ash, 3% crude fiber, and 6.5% moisture. The granule size is 0.36–0.65 mm and the energy density is 3.39 kcal/g. Otohime B2 is available online outside Japan (e.g. USA or UK) (http://www.reedmariculture.com/product_otohime_fish_diet.php). Body weights and fasting blood glucose were measured weekly^27^ and plasma triglycerides were analysed once every 2 weeks as described previously^25^.

### Glucose tolerance test

The intraperitoneal glucose tolerance test (IPGTT) was performed as described previously^28^. Fish were anesthetized using ice water (gradually from 17 °C to 12 °C) for approximately 5 min, injected intraperitoneally with 0.5 mg glucose/g fish weight and allowed to recover for 30, 90, and 180 min after injection. Blood was collected and blood glucose was determined at each time point^25, 29^. For the oral glucose tolerance test (OGTT), a micropipette with a small tip was gently inserted into the mouth of the anesthetized fish, and a glucose solution was administered at a dose of 1.25 mg/g fish weight. Fish were allowed to recover in the water system for 30, 60, and 120 min after dosing, and blood samples were collected at each time point to determine the blood glucose levels.

### Ins-EGFP DIO zebrafish image analysis

Ins-EGFP zebrafish were overfed as described above for 3 months, then fasted overnight and anesthetized by placing them in a tank containing 500 ppm of 2-phenoxyethanol (Wako Pure Chemicals, Osaka, Japan). EGFP signalling was observed and images captured using an Olympus SZX7 microscope with GFP filter (Olympus, Tokyo, Japan). The EGFP-positive intensity was quantified using ImageJ software (National Institutes of Health, Bethesda, MD, USA). Images were acquired at identical settings. Individual images were imported in ImageJ and converted to grayscale after RGB splitting the images to extract green (EGFP) signals. The average fluorescence intensity of pancreatic area was quantified as mean pixel density according to a previous study^30^. The EGFP positive area was converted to a percentage of the entire image and the relative EGFP intensity was calculated by normalizing to the average EGFP intensity of the non-DIO group.

### Western blot analysis

Liver-pancreas tissues were isolated from normal and overfed ins-EGFP zebrafish. A rabbit polyclonal antibody to GFP (GeneTex, Irvine, CA, USA) or anti-GAPDH (AnaSpec, San Jose, CA, USA) was used and detected via chemiluminescence. Additional methods details are given in Supplementary Materials and Methods.

### Metformin and glimepiride administration

DIO zebrafish were administered metformin (Enzo Life Sciences, Farmingdale, NY, USA) and glimepiride (LKT Laboratories, St. Paul, MN, USA) as described previously with the following modifications^31^. In brief, we dissolved metformin in fish water to a final concentration of 20 µM. The metformin solution was freshly prepared and changed daily. Blood samples were collected after 7 days of metformin exposure. Glimepiride was dissolved in DMSO to make a 500 mM stock solution, then diluted to 100 µM in fish water. Blood samples were collected after 24 h of glimepiride exposure.

### RNA isolation, library construction and high-throughput sequencing

After 8 weeks of overfeeding, zebrafish were sacrificed for harvesting of liver-pancreas tissues and total RNA was isolated and purified using Isogen (Nippongene, Tokyo, Japan) combined with the RNeasy mini kit (Qiagen, Hilden, Germany). Ribosomal RNA was depleted using the Ribo-Zero Magnetic Kit (Epicentre, Madison, WI, USA) according to the manufacturer’s protocol. RNA library construction was then performed by The Center of Genetics (Mie University, Japan) using the Ion Total RNA-Seq Kit v2 (Life Technologies, Carlsbad, USA) following the manufacturer’s instructions (4476290, revision C). Preparations containing bar-coded libraries were loaded into 318 Chips and sequenced on the Ion PGM system (Life Technologies). Additional methods details are outlined in Supplementary Materials and Methods.

### Bioinformatics analysis of RNA-Seq data

Dual RNA-seq data were mapped and tag counts were performed using Genomics Workbench (CLC bio, Aarhus, Denmark). We then performed gene expression analysis using the R package and Tag Count Data Comparison (TCC). This package incorporates multi-step normalization methods, whose strategy is to remove potential genuine differentially expressed genes before performing data normalization^32^. After statistical tests, we performed comparative genomics analyses (Gene-Set Enrichment Analysis [GSEA] and Sub-network Enrichment Analysis [SNEA]) using Pathway Studio 9.0 (Elsevier, Amsterdam, Holland) according to our previous study^33^.

### Statistical analysis

All results are presented as means with their standard errors (SE). Data ware analysed using Student’s t-test or ANOVA with the Bonferroni–Dunn multiple comparison procedure, depending on the number of comparisons. A *P*-value of less than 0.05 was considered statistically significant.

## Results

### Diet-induced obesity model of zebrafish with high fasting blood glucose

**Figure 1:**
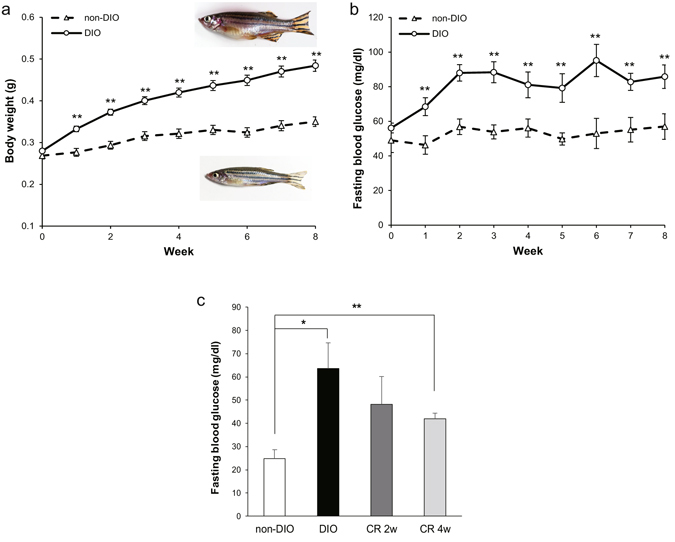
Body weights (g), fasting blood glucose of overfed (DIO) and normal-fed (non-DIO) zebrafish, and effect of calorie restriction on DIO zebrafish. (**a**) DIO zebrafish gain weight progressively over an 8-week period. Representative images of DIO (upper) and non-DIO (lower) zebrafish are shown in the panel. Non-DIO group: *n* = 14; DIO group: *n* = 29. (**b**) Changes in the fasting blood glucose concentrations of the non-DIO and DIO groups. Non-DIO group: *n* = 14; DIO group: *n* = 29. (**c**) Changes in fasting blood glucose concentrations after calorie restriction for 2 and 4 weeks. *n* = 5–8. Values are means ± SE. *P < 0.05, **P < 0.01 vs. the non-DIO group.

It was previously reported that development of DIO in zebrafish requires no less than 150 calories from freshly hatched *Artemia* ^15^. To create DIO zebrafish with hyperglycaemia, we developed a new feeding method by overfeeding the fish with a commercial fish food, Otohime B2, using an automated feeding system. The quantity of diet fed to the overfed group (DIO) was six times that fed to the control group, providing 408 vs. 68 calories per fish per day, respectively. After 1 week of overfeeding, the body weight of the DIO group was significantly (*P* < 0.01) increased compared with the non-DIO group (Fig. 1a). At the same time, the fasting blood glucose (FBG) of the DIO group (68 ± 5 mg/dl) was already significantly (*P* < 0.01) higher than that of the control group (46 ± 5 mg/dl), and this trend continued until the end of the study (Fig. 1b). The visceral adipose tissue volume, plasma triglyceride, and lipid accumulation in liver (Supplementary Fig. S1) were also increased in the DIO group compared with the non-DIO group, consistent with their obese phenotypes.

Since calorie restriction (CR) is the most frequently prescribed treatment for T2DM, we overfed zebrafish for 8 weeks as described above before restricting them to 20 mg per fish per day of Otohime B2, fed once daily (the same feeding regime as the non-DIO group) for 4 weeks. Compared to the start of CR, the FBG was decreased by 24% after 2 weeks of CR (from 64 ± 11 to 48 ± 12 mg/dl), and was further decreased by 34% after 4 weeks of CR (42 ± 2 mg/dl). However, the FBG levels were still significantly higher than that of the non-DIO group (*P* < 0.01) and were not significant different from the DIO group (Fig. 1c).

### Glucose intolerance with insulin overproduction in DIO zebrafish

**Figure 2:**
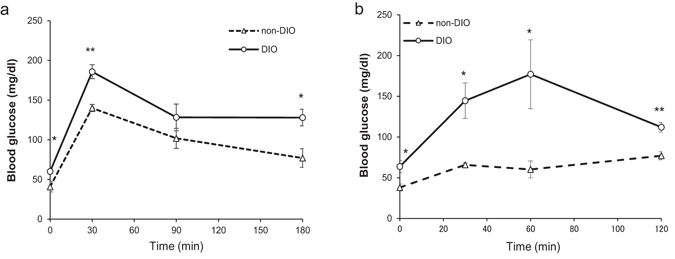
Impaired glucose tolerance in DIO zebrafish. (**a**) Intraperitoneal glucose tolerance test (IPGTT) in non-DIO and DIO zebrafish with blood glucose levels determined after fasting (0 min) and 30, 90, and 180 min after intraperitoneal injection of 0.5 mg glucose/g body weight; *n* = 5 fish/time point. (**b**) Oral glucose tolerance test (OGTT) in non-DIO and DIO zebrafish with blood glucose levels determined after fasting (0 min) and 30, 60, and 120 min after oral administration of 1.25 mg glucose/g body weight; *n* = 5 fish/time point. Values are means ± SE. *P < 0.05, **P < 0.01 vs. the non-DIO group.

To confirm our DIO zebrafish as a model for impaired glucose tolerance, we performed intraperitoneal and oral glucose tolerance tests (IPGTT and OGTT). For the IPGTT, we injected fish with 0.5 mg glucose/g body weight, and examined the blood glucose (BG) concentrations at 0, 30, 90, and 180 min after injection (Fig. 2a). The BG of the non-DIO group peaked at 30 min after injection, and gradually recovered to basal levels (before glucose injection) by 180 min. DIO zebrafish exhibited a similar glucose curve, however their BG levels were significantly higher than those of the controls at 30 and 180 min after injection and did not recover to basal levels during the test. For the OGTT, a glucose solution was orally administered to fish at a dose of 1.25 mg/g body weight, and BG was measured at 0, 30, 60, and 120 min after administration. As shown in Fig. 2b, the BG level of the DIO group showed a marked increase compared with the non-DIO group, peaked at 60 min, and was significantly higher than that of the control group at all-time points. In addition, the postprandial glucose level of the DIO group increased compared with the non-DIO group (Supplementary Fig. S2).

**Figure 3:**
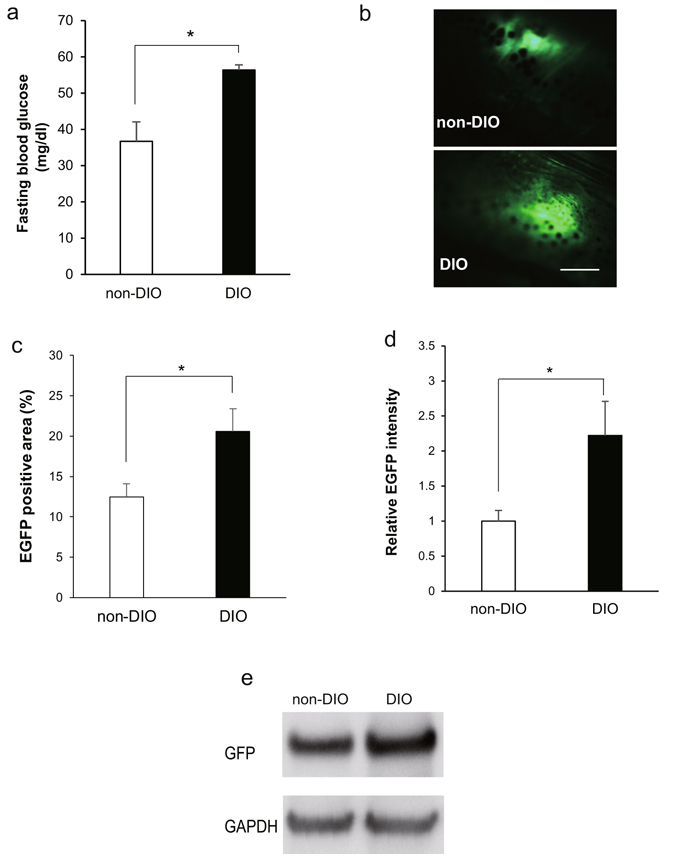
Increased insulin production in DIO zebrafish. (**a**) Fasting blood glucose concentrations of ins-EGFP zebrafish after 3 months of normal feeding or overfeeding. *n* = 10. (**b**) Insulin-EGFP signals in exocrine pancreas were monitored in non-DIO and DIO ins-EGFP zebrafish by fluorescence stereoscopic microscopy. Scale bar = 0.5 mm. (**c**) Graph of the percentage of EGFP positive signal area in entire image. *n* = 3. (**d**) Graph of relative EGFP intensities from panel b. *n* = 3. (**e**) Western blot analysis for GFP signals in liver-pancreas tissue of non-DIO and DIO ins-EGFP. Full-length blots are presented in Supplementary Fig. S3. Values are means ± SE. *P < 0.05, **P < 0.01 vs. the non-DIO group.

Since impaired glucose tolerance is a pre-diabetic state of hyperglycaemia that is associated with insulin resistance, we quantified the volume of insulin secreted by DIO zebrafish. Because there is no commercially-available antibody for zebrafish insulin suitable for western blot analysis, we used *Tg(−1.0ins:EGFP)* ^*sc1*^ transgenic zebrafish (ins-EGFP) and quantified insulin levels by EGFP expression. In ins-EGFP zebrafish, EGFP expression is driven by the zebrafish preproinsulin promoter, so insulin-expressing cells of the pancreatic islets were visualised^34^. After 3 months of overfeeding, ins-EGFP DIO zebrafish showed significantly (*P* < 0.05) higher FBG (56 ± 1 mg/dl) than the ins-EGFP non-DIO group (37 ± 5 mg/dl; Fig. 3a). We monitored EGFP expression in the pancreas of these zebrafish using fluorescence stereoscopic microscopy (Fig. 3b). The EGFP positive area in pancreas region was significantly increased in the DIO group (*P* < 0.05, Fig. 3c) and the average intensity of EGFP signals were increased 2.2-fold compared with non-DIO zebrafish (Fig. 3d). Western blot analysis revealed that the abundance of GFP protein in DIO zebrafish was higher than that of the non-DIO group (Fig. 3e), consistent with EGFP fluorescence. These results indicate that DIO zebrafish suffered from impaired glucose tolerance with increased insulin production, consistent with an insulin resistance model for T2DM.

### Anti-diabetic drugs reduce blood glucose levels of DIO zebrafish

**Figure 4:**
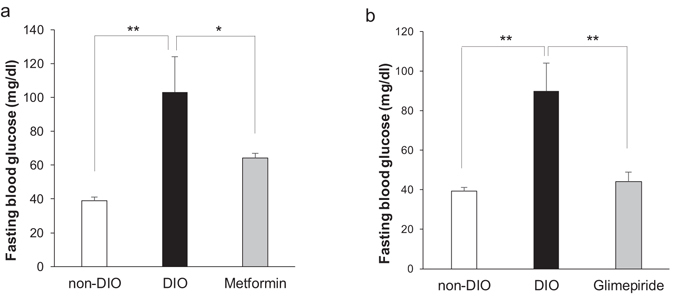
The effects of anti-diabetic drugs on DIO zebrafish. (**a**) Effect of metformin (20 μM) on blood glucose levels of DIO zebrafish after 7 days’ treatment. (**b**) Effect of glimepiride (100 μM) on blood glucose levels of DIO zebrafish after 24 h treatment. *n* = 5–10. Values are means ± SE. *P < 0.05, **P < 0.01 vs. the non-DIO group.

To validate that DIO zebrafish can be used to predict the human response to drugs and other chemicals, we administered the anti-diabetic drugs metformin and glimepiride. After 7 days of metformin exposure (final concentration 20 µM), the blood glucose of DIO zebrafish was significantly (*P* < 0.05) decreased compared with that of untreated DIO zebrafish (64 ± 3 mg/dl vs. 90 ± 14 mg/dl; Fig. 4a). Glimepiride exposure (final concentration 100 µM) for 24 h also significantly (*P* < 0.01) reduced the blood glucose levels of DIO zebrafish (to 44 ± 5 mg/dl; Fig. 4b).

### Gene expression profiling of liver-pancreas revealed pathways common to human T2DM

To investigate the transcriptional mechanism for the glucose intolerance in DIO zebrafish, we conducted RNA deep sequencing (RNA-Seq) of liver-pancreas tissues. RankProd analysis^35^ identified 83 and 64 genes as significantly (false discovery rate [FDR] < 0.2) increased and decreased, respectively, in DIO zebrafish compared with control zebrafish (Supplementary Table S1). According to the Ensembl gene orthologs database (http://www.ensembl.org/biomart/martview), these 147 zebrafish genes corresponded to 121 human orthologs. We conducted Gene Ontology (GO) Enrichment Analysis of these altered genes in the biological process category. GOs related to circadian rhythm, response to oxygen/stress, transcriptions, and nitrogen metabolism were enriched in these altered genes (Table 1).

**Table 1:** Gene Ontology (GO) Enrichment Analysis of altered genes categorized by biological process.

Category	GO biological process	GO accession	P-value
Circadian rhythm	entrainment of circadian clock	GO:0009649	3.14E-03
	circadian regulation of gene expression	GO:0032922	1.42E-05
Response to oxygen levels	cellular response to oxygen levels	GO:0071453	1.83E-02
Response to stress	response to abiotic stimulus	GO:0009628	1.44E-02
	cellular response to stress	GO:0033554	1.57E-02
	cellular response to chemical stimulus	GO:0070887	6.21E-03
	response to organic substance	GO:0010033	1.23E-02
Transcription	transcription, DNA-templated	GO:0006351	2.61E-02
	nucleic acid-templated transcription	GO:0097659	2.64E-02
	RNA metabolic process	GO:0016070	7.83E-04
Nitrogen metabolism	cellular nitrogen compound metabolic process	GO:0034641	6.33E-05
	nitrogen compound metabolic process	GO:0006807	1.67E-05

**Figure 5:**
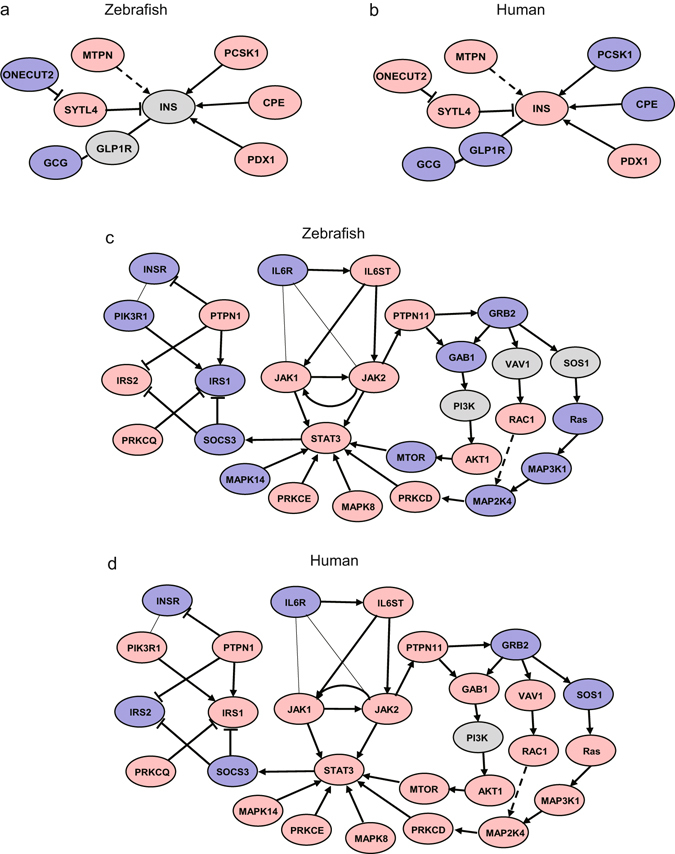
Pathways of insulin secretion in pancreas and insulin resistance in liver of DIO zebrafish and T2DM patients. Insulin secretion pathways in (**a**) DIO zebrafish and (**b**) pancreatic beta cells of T2DM human patients (GSE20966). Pathways of insulin resistance in (**c**) DIO zebrafish and (**d**) human hyperglycaemic liver (GSE23343). Red and blue denote genes with increased and decreased expression, respectively. Grey denotes genes that were not detected in the RNA-seq assay or DNA microarray.

To investigate whether DIO zebrafish share transcriptomic pathways common to human diabetes, we conducted Pathway Analysis^36^ and Gene-Set Enrichment Analysis (GSEA)^37^. DNA microarray data of GSE20966 pancreatic beta cells^38^ and GSE23343 hyperglycaemic liver^39^ of T2DM patients were selected as reference gene expression profiles. For the Pathway Analysis, pathways of insulin secretion in pancreas and insulin resistance in liver are depicted in Fig. 5. The insulin secretion pathway is similar between DIO zebrafish and T2DM patients, while the insulin resistance pathway shows differences, especially in the RAS – MAP3K cascade. Next, we conducted GSEA to identify gene-sets common to DIO zebrafish and T2DM patients. GSEA can determine which gene-sets (a priori defined sets of genes belonging to the same biological pathway) tend to occur in the gene list generated between two biological states. For the diabetic pancreas, gene-sets related to atherosclerosis, nervous system, immune response and vascularization, circadian rhythm and melanoma-related proteins were listed as common to human and zebrafish T2DM (Table 2). For the diabetic liver, gene-sets related to immune response, nervous system and vascularization were listed as common to human and zebrafish T2DM, similar to the pancreas results. In addition, MAP kinase activity, oxygen transport and voltage-gated calcium channel activity were also listed for the diabetic liver as gene-sets common to human and zebrafish T2DM (Table 3).

**Table 2:** Gene-sets common to the hepatopancreas of DIO zebrafish and pancreatic beta cells of human type 2 diabetes patients (GSE20966) according to Gene-Set Enrichment Analysis (GSEA) analysis.

Category	Name	*P* value	
		DIO-zebrafish	GSE20966
Obese	Proteins Involved in Pathogenesis of Atherosclerosis	7.E-04	7.E-03
Immune response	Chemotaxis	2.E-05	2.E-05
	Mast Cells de Novo Synthesized Mediators via IgE Independent Signaling	2.E-02	2.E-03
	Immune response	2.E-02	5.E-04
Nervous system	Memory	4.E-03	2.E-03
	Synaptic transmission	6.E-04	2.E-02
Vascularization	Angiogenesis	2.E-02	9.E-10
Other	Regulation of Circadian Clock Genes in Suprachiasmatic Nuclei Neurons	2.E-02	9.E-03
	Proteins Involved in Pathogenesis of Melanoma	1.E-02	8.E-03

**Table 3:** Gene-sets common to the hepatopancreas of DIO zebrafish and hyperglycaemic liver of human type 2 diabetes mellitus patients (GSE23343) according to Gene-Set Enrichment Analysis (GSEA) analysis.

Category	Name	*P* value	
		DIO-zebrafish	GSE23343
Immune response	Humoral immune response	4.36E-03	9.77E-03
	B cell apoptotic process	1.07E-02	2.61E-02
Nervous system	Synaptic transmission	6.26E-04	8.82E-07
	Positive regulation of synapse maturation	1.74E-02	2.11E-02
Vascularization	Artery morphogenesis	2.39E-02	2.50E-02
	Homophilic cell adhesion via plasma membrane adhesion molecules	3.53E-02	5.14E-06
	Positive regulation of epithelial cell proliferation	3.62E-02	2.13E-02
Other	Positive regulation of MAP kinase activity	8.02E-04	1.86E-02
	Oxygen transport	4.55E-02	1.60E-02
	Regulation of voltage-gated calcium channel activity	3.E-02	1.E-02

## Discussion

The pathogenesis of obesity-related insulin resistance is a complex and multifactorial process involving the sequential interplay of several tissues, including pancreas, liver, skeletal muscle and adipose tissue. For pancreas, zebrafish β-cells are similar to those of mammals in development and function in maintaining glucose homoeostasis^40^, and almost all zebrafish orthologs (except *ngn3*) are functionally conserved during β-cell development^41^. In this study, we developed a zebrafish T2DM model by overfeeding. The hyperglycaemia persists even after 4 weeks of calorie restriction (Fig. 1c), which indicates that the hyperglycaemia in this T2DM model is irreversible and not a transient phenomenon. We demonstrate that over-nutrition leads to increased β-cell mass in our T2DM zebrafish (Fig. 3b–d), which may result from compensatory beta cell hypertrophy and hyperplasia in response to hyperglycaemia in diabetogenic states similar to rodents^42^ and humans^43^, indicating insulin resistance in these zebrafish.

In particular, visceral adipose tissue (VAT) accumulation plays an important role. Studies have demonstrated that excess VAT is associated with decreased sensitivity of glucose uptake to insulin stimulation, reduced free fatty acid re-esterification rate, and increased lipolysis resistance to the inhibitory effect of insulin in both visceral and peripheral adipocytes^44^. T2DM zebrafish showed increased insulin release with VAT accumulation and hepatic steatosis (Supplementary Fig. S1a and c), which suggests that zebrafish T2DM develops simultaneously and synergistically in multiple interacting organs.

For drug response, as an animal model, T2DM zebrafish were responsive to the anti-diabetic drugs metformin and glimepiride (Fig. 4). Metformin is recommended as the first line drug and is the only pharmacologic agent recommended for the prevention or delay of T2DM in at-risk subjects^45^. Metformin enhances the action of insulin by increasing translocation of glucose transporters^46^, increasing the activity of AMP kinase^47^, or reducing the activity of DPP4^48^. Glimepiride is a second-generation sulfonylurea that promotes the induction of ATPase-dependent potassium channels in β-cells of the pancreas, stimulating insulin release^49^. These results suggest that T2DM zebrafish may respond to other anti-diabetic drugs, allowing for zebrafish screening of new drugs.

To evaluate the similarity of pathological transcriptome pathways between T2DM zebrafish and human T2DM patients, we conducted bioinformatics analysis of these transcriptome profiles. GO analysis revealed that a gene-set related to circadian rhythm was altered in T2DM zebrafish. Growing evidence indicates that disruption of our internal timing system contributes to the incidence and severity of metabolic diseases, including obesity and T2DM. For example, circadian disruption and DIO synergistically promote development of pancreatic β-cell failure and diabetes in male rats^50^. In addition, some variants of the clock circadian regulator genes have been associated with the prevalence of T2DM^51^. In leptin-resistant Zucker diabetic fatty rats, the clock genes were altered in a tissue-dependent manner, including pancreas^52^. Moreover, Angiopoietin-like 2, a circadian gene, improves T2DM through potentiation of insulin sensitivity in mice^53^, suggesting that our GO results imply possibility for the circadian genes to act as drug targets against T2DM.

Because the zebrafish has a hepatopancreas that combines the functions of pancreas and liver, we compare their hepatopancreas RNA-seq data to those of human pancreatic β cells and liver of T2DM patients using GSEA. The GSEA results showed that pathways related to immune response, nervous systems and vascularization were common to pancreas (Table 2) and liver (Table 3) in both species. These are strong results; however, the pathway related to obesity (Atherosclerosis in Table 2) was detected only in pancreas, not in liver. In relation to the conventional pathways in T2DM, pathways of insulin secretion in pancreas showed similar gene expression alterations between zebrafish and human (Fig. 5a, b), while pathways of insulin resistance in liver (Fig. 5c, d) showed species similarity only in the central cascade, IL6 – STAT3, a known pathway in human T2DM^54^. The difference in peripheral pathways between zebrafish and humans implies that the biological function of some non-hub proteins in zebrafish differs from that of human, especially the RAS – MAP3K pathway. These issues require close investigation for understanding the comparative and functional genomics in zebrafish. The comparative transcriptome analysis revealed that T2DM zebrafish and human T2DM patients share common pathological pathways. These findings suggest that the T2DM zebrafish model can be used to identify putative pharmacological targets and to test novel drugs for treatment of human obesity.

In addition, we demonstrate that 1 week of overfeeding is enough to induce T2DM in zebrafish. This is short compared with mammals, who typically take 2 months to develop the T2DM phenotypes of glucose intolerance, insulin resistance, and enhanced β-cell mass and proliferation^55–57^. On the effects of feeding a high-fat diet (HFD) for a short term (less than 4 weeks) in mice, Rockann E.M. *et al*. reported HFD-induced β-cell proliferation after 3 days and slight glucose intolerance after 7 days^58^. Turner N. *et al*. also reported for mice that glucose intolerance developed within 3 days of HFD feeding, but FBG was not significantly elevated until 6 weeks of HFD feeding^57^. One week of overfeeding enhanced FBG levels in zebrafish to approximately 1.5-fold those of normal feeding. This suggests that the zebrafish is more susceptible to glucose intolerance than the mouse. We hypothesize that this may be caused by the dietary habits of zebrafish. In nature, the diet of this species consists of other animals like benthic and planktonic crustaceans, worms and insect larvae, and is less in carbohydrates^59^. For this reason, the glucose tolerance of zebrafish may be more affected by an overload of carbohydrate than that of mammals, implying zebrafish may be more susceptible to T2DM.

## Conclusions

In this study, we created a zebrafish model of T2DM using a method of overfeeding. T2DM zebrafish exhibited increased FBG with glucose intolerance and insulin resistance. There are several advantages to our T2DM zebrafish model: 1) rapid onset of T2DM phenotypes compared with rodent models, 2) positive response to human anti-diabetic drugs using exposure or oral administration methods we developed previously^27^, and 3) pathological similarity in transcriptomic pathways to the human platform. Our findings suggest that T2DM zebrafish are suitable for elucidating the mechanisms of early phase T2DM and will prove to be a useful animal model for phenotype-driven drug discovery against diabetes.

## Electronic supplementary material

Supplementary Information
