# Supplementary material for: Development of a Novel Zebrafish Model for Type 2 Diabetes Mellitus
Source: Sci Rep. 2017 May 3;7:1461. doi: 10.1038/s41598-017-01432-w (PMC5431185; doi:10.1038/s41598-017-01432-w)
Supplement: Supplementary file 1 — Supplementary Information [file 41598_2017_1432_MOESM1_ESM.pdf]

Development of a Novel Zebrafish Model for Type 2 Diabetes Mellitus

Liqing Zang[#1], Yasuhito Shimada[#2, 3], Norihiro Nishimura[1]

[1]Graduate School of Regional Innovation Studies, Mie University, Tsu, Mie, Japan

[2]Department of Molecular and Cellular Pharmacology, Pharmacogenomics, and Pharmacoinformatics, Mie University Graduate School of Medicine, Tsu, Mie, Japan

[3]Department of Bioinformatics, Mie University Life Science Research Center

[#] These authors contributed equally to this work.

Correspondence: Dr. Liqing Zang, Graduate School of Regional Innovation Studies, Mie University, 1577 Kurimamachiya-cho, Tsu, Mie, Japan

Tel. /fax: +81 59 231 5405

E-mail: liqing@doc.medic.mie-u.ac.jp (L. Zang)

Running title: Diet-induced Type 2 Diabetes Mellitus in Zebrafish

Keywords: overfeeding; hyperglycaemia; glucose intolerance; insulin resistance; RNA-seq; zebrafish

Supplementary Materials and Methods

CT measurement of visceral adipose tissue volume

Zebrafish were euthanized by immersion in an ice-water bath (5 parts ice/1 part water at ≤ 4 °C) for ≥ 20 min[1] and a 3D micro-CT scan was performed using an in vivo System R_mCT 3D micro-CT scanner (Rigaku, Tokyo, Japan) as described previously[2]. 3D images were reconstructed and viewed using i-View type R software (J. Morita Mfg, Kyoto, Japan), and visualized and analysed using CT Atlas Metabolic Analysis ver. 2.03 software (Rigaku). Measurement of visceral adipose tissue volume was limited to the abdominal cavity, defined as the area between the cleithrum and the anus.

2-hour postprandial glucose test

Fish were given ad libitum access to Otohime B2 for 5 min. The food was then removed, and after 30, 60, and 120 min, fish were anesthetized using ice water, a blood sample was collected and the blood glucose level was determined.

Western Blot analysis

Normal-fed and overfed ins-EGFP zebrafish were euthanized and the abdominal wall was stripped off. The intra-abdominal organs were excised as a whole and rapidly transferred to a petri-dish filled with pre-chilled saline. Under a stereomicroscope (Nikon, Tokyo, Japan), liver-pancreas tissues were isolated with fine scissors and forceps and weighed. T-PER Tissue Protein Extraction Reagent (Thermo Scientific, Rockford, IL) with a protease inhibitor cocktail (Thermo Scientific) was then added to tissues at a ratio of 18 μl buffer/1 mg tissue. After homogenization, protein samples were centrifuged at 20 000 × g for 20 min at 4 °C. The concentration of each protein sample was measured using Pierce 660 nm Protein Assay Reagent (Thermo Scientific). For Western blot analysis, protein samples were separated by 12% sodium dodecyl sulphate-polyacrylamide gel electrophoresis, transferred onto a polyvinylidene fluoride (PVDF) membrane (Life Technologies), and blocked at 4 °C with PVDF Blocking Reagent (Toyobo, Osaka, Japan) overnight. The membrane was incubated with Rabbit Polyclonal antibody to GFP (1:20 000; GeneTex, CA, USA) or anti-glyceraldehyde-3-phosphate dehydrogenase (GAPDH) Z-Fish (1:3000; ANASpec, San Jose, CA) for 1 h, and then washed with TBS containing 0.1% Tween-20 (TBST) three times. The membrane was then incubated in Goat Anti-Rabbit IgG H&L (HRP) (1:10 000 or 20 000, Abcam, Cambridge, MA) for 1 h. ImmunoStar Zeta (Wako Pure Chemicals, Osaka, Japan) was used for detection. Membranes were imaged and quantified with an

ImageQuant LAS4000 instrument using the ImageQuantTL software (GE Healthcare, Waukesha, WI).

RNA isolation, library construction and high-throughput sequencing

Fish were given a laparotomy, immediately transferred into tubes containing 3 mL of RNAlater (Qiagen, Hilden, Germany) and placed at 4 °C for 24 h, and then stored at –20 °C until use. The entire hepatopancreas was isolated by dissection using forceps. RNA was isolated from the hepatopancreas using Isogen (Nippongene, Tokyo, Japan) combined with the clean-up protocol of the RNeasy mini kit (Qiagen). Total RNA content was measured using the Nano Drop 1000 (Thermo Fisher Scientific, Waltham, MA, USA), and the integrity of RNA samples was determined using an Agilent 2100 Bioanalyzer RNA 6000 Pico Chip (Agilent Technologies, Santa Clara, CA, USA).

Before library construction, ribosomal RNA was depleted from 5 μg high quality (RIN > 8.0) RNA samples using a Ribo-Zero Magnetic Kit (Epicentre, Madison, WI, USA) according to the manufacturer's protocol. The integrity of the rRNA-depleted RNA was confirmed using the Agilent RNA Pico assay reagents and Bioanalyzer 2100 system (Agilent Technologies). RNA library construction was then performed by The Center of Genetics (Mie University, Japan) using an Ion Total RNA-Seq Kit v2 (Life Technologies, Carlsbad, USA) following the manufacturer's instructions (4476290, revision C). Briefly, the mRNA was fragmented using RNaseIII and purified. The fragmented RNA was hybridized and ligated with the Ion adaptor. The RNA fragments were reverse transcribed and amplified to cDNA. The amplified cDNA was purified using a magnetic bead-based method, and the molar concentration was determined for each cDNA library. Emulsion PCR and enrichment steps were carried out using an Ion OneTouch 200 template kit v2 DL as described in the manufacturer's protocol (MAN0006957, revision 5.0). Assessment of the Ion Sphere particle quality was undertaken between the emulsion PCR and enrichment steps with an Ion Sphere quality control kit using a Qubit 2.0 fluorometer (Life Technologies). Preparations containing bar-coded libraries were loaded into 318 Chips and sequenced on the PGM system (Life Technologies). Signal processing and base calling were performed with Torrent Suite software version 4.0.1. Adapter sequences were trimmed using the same software.

Supplementary Figures

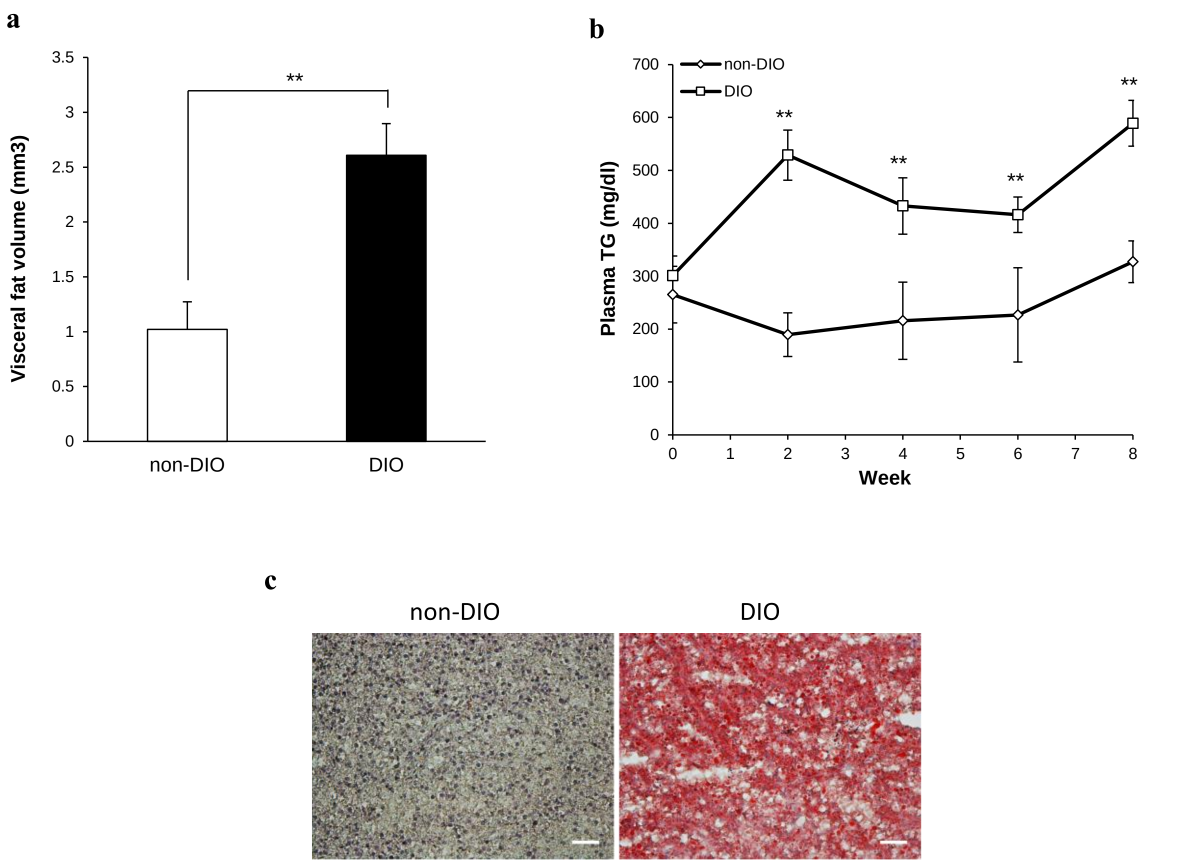

Supplementary Figure S1. Assessment of visceral adipose tissue volume, plasma TG and hepatic steatosis in overfed zebrafish. (a) Changes in visceral adipose tissue volume in non-DIO and DIO zebrafish. Non-DIO group: $n = 14$; DIO group: $n = 29$. (b) Changes in plasma triglyceride (TG) levels in non-DIO and DIO zebrafish. Non-DIO group: $n = 14$; DIO group: $n = 29$. (c) Oil red O staining of liver sections. Red droplets indicate neutral lipid staining. Original magnification: × 400. Scale bar = 50 µm. Values are means ± SE. Statistical analyses were performed using Student's t-test to compare the non-DIO and DIO groups at each time-point. *$P < 0.05$, **$P < 0.01$.

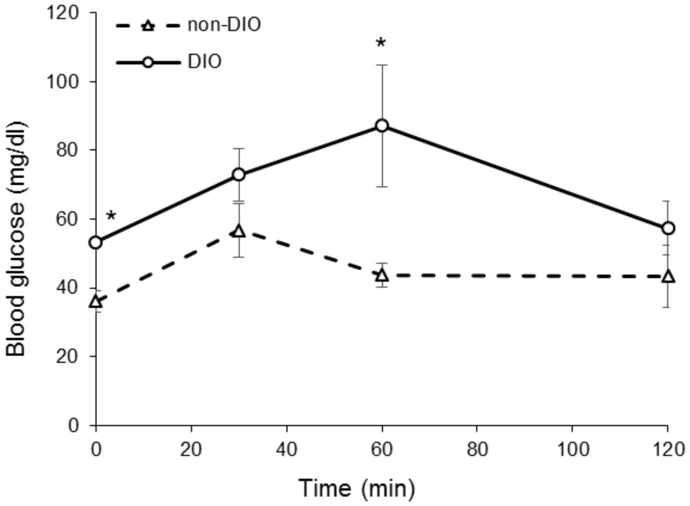

Supplementary Figure S2. Postprandial glucose tolerance test. Blood glucose levels were determined after fasting (0 min) and 30, 60, and 120 min after food intake in non-DIO and DIO zebrafish; $n = 5$ fish/time point. Values are means ± SE. * $P < 0.05$, ** $P < 0.01$ vs. the non-DIO group.

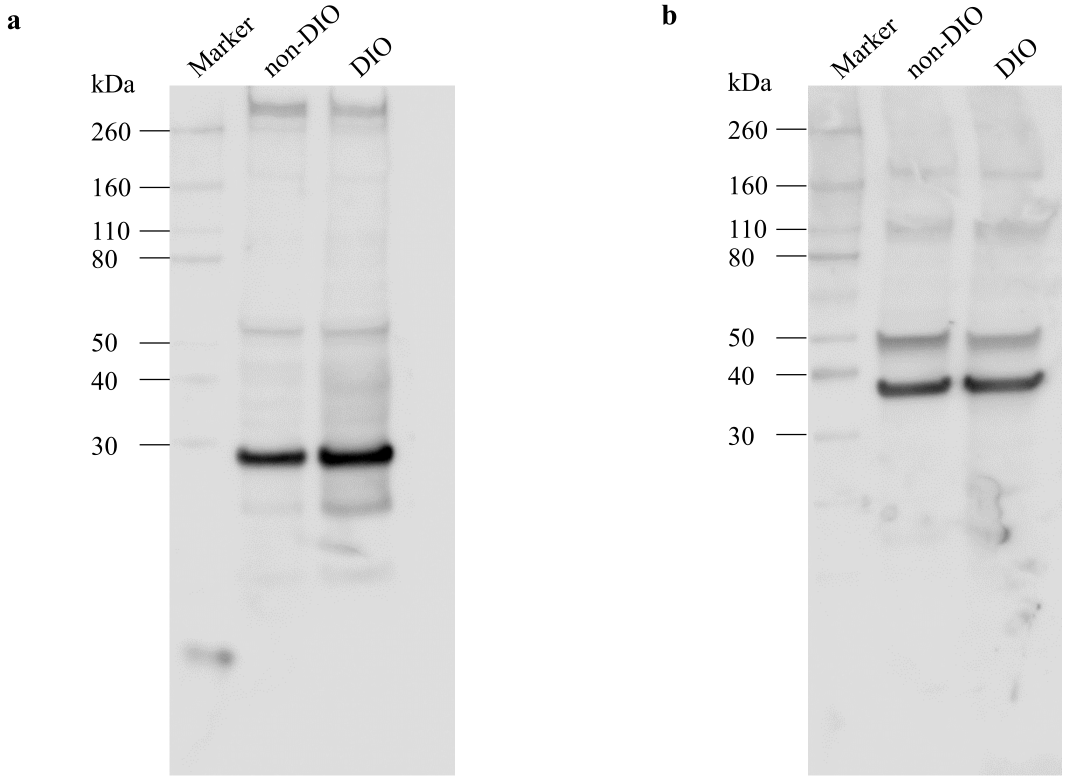

Supplementary Figure S3. Western blot analysis. Full-length Western blot images for GFP (a) and GAPDH (b) signals in liver-pancreas tissue of non-DIO and DIO ins-EGFP. The predicted GFP and GAPDH target sizes are 29 kDa and 36 kDa, respectively.

Table S1. Genes dysregulated in DIO zebrafish compared with non-DIO zebrafish.

Zebrafish		Human		Normalized tag count				
Feature ID	Gene ID	Gene Symbol	Ortholog ID	Control	T2DM	Log ratio	p.value	q.value
si:dkey-93m18.6	ENSDARG00000093173	-	-	45.6	2.3	-4.3	0.0	0.01
tas1r3	ENSDARG00000006341	TAS1R3	ENSG00000169962	18.2	1.0	-4.2	0.0	0.05
pros1	ENSDARG00000068261	PROS1	ENSG00000184500	25.1	1.4	-4.1	0.0	0.00
si:ch211-209j12.2	ENSDARG00000099337	-	-	34.0	2.1	-4.0	0.0	0.01
si:dkeyp-98a7.8	ENSDARG00000094674	-	-	19.5	1.6	-3.6	0.0	0.08
gadd45ba	ENSDARG00000027744	GADD45B	ENSG00000099860	778.2	77.4	-3.3	0.0	0.00
per1b	ENSDARG00000012499	PER1	ENSG00000179094	474.8	67.7	-2.8	0.0	0.00
si:ch211-195h23.3	ENSDARG00000068431	-	-	92.9	13.5	-2.8	0.0	0.02
syn1	ENSDARG00000060368	SYN1	ENSG00000008056	30.3	4.6	-2.7	0.0	0.04
sik1	ENSDARG00000058606	CH507-42P11.8	ENSG00000275993	534.8	105.1	-2.3	0.0	0.07
pcxb	ENSDARG00000051939	PC	ENSG00000173599	145.6	29.5	-2.3	0.0	0.12
MYOCD	ENSDARG00000098836	MYOCD	ENSG00000141052	49.4	10.6	-2.2	0.0	0.15
BX005085.6	Transcription	ZNF260	ENSG00000254004	40.3	8.7	-2.2	0.0	0.02
TC2N	ENSDARG00000095067	TC2N	ENSG00000165929	22.6	5.0	-2.2	0.0	0.16
pou6f2	ENSDARG00000086362	POU6F2	ENSG00000106536	175.2	38.7	-2.2	0.0	0.17
cyp7a1a	ENSDARG00000069018	CYP7A1	ENSG00000167910	1473.6	328.5	-2.2	0.0	0.01
si:ch211-277e21.1	ENSDARG00000096800	-	-	39.1	8.8	-2.1	0.0	0.07
znf395b	ENSDARG00000024195	ZNF395	ENSG00000186918	86.8	20.7	-2.1	0.0	0.00
cyp2ad6	ENSDARG00000042956	CYP2J2	ENSG00000134716	132.1	32.0	-2.0	0.0	0.05
si:dkey-186o21.1	ENSDARG00000099633	SEC14L3	ENSG00000100012	312.3	77.9	-2.0	0.0	0.07
ppp1r3ca	ENSDARG00000071005	PPP1R3C	ENSG00000119938	2220.1	574.4	-2.0	0.0	0.03
cry1aa	ENSDARG00000011583	CRY1	ENSG00000008405	288.8	75.7	-1.9	0.0	0.00
cry2	ENSDARG00000011890	-	-	257.0	67.3	-1.9	0.0	0.00
zgc:103755	ENSDARG00000104663	CLIP4	ENSG00000115295	34.7	9.1	-1.9	0.0	0.10
mycb	ENSDARG00000007241	MYC	ENSG00000136997	593.0	157.5	-1.9	0.0	0.04

sik2a	ENSDARG00000079618	SIK2	ENSG00000170145	57.9	15.6	-1.9	0.0	0.15
per3	ENSDARG00000010519	PER3	ENSG00000049246	535.1	144.2	-1.9	0.0	0.00
tefa	ENSDARG00000039117	TEF	ENSG00000167074	698.2	200.4	-1.8	0.0	0.06
bhlhe40	ENSDARG00000004060	BHLHE40	ENSG00000134107	628.1	181.5	-1.8	0.0	0.04
mxd1	ENSDARG00000032039	MXD1	ENSG00000059728	52.9	15.3	-1.8	0.0	0.16
mtss1la	ENSDARG00000028943	MTSS1L	ENSG00000132613	1289.0	384.8	-1.7	0.0	0.01
cry5	ENSDARG00000053502	CRYAA	ENSG00000160202	98.4	30.1	-1.7	0.0	0.05
plcd3a	ENSDARG00000052957	PLCD3	ENSG00000161714	88.7	27.5	-1.7	0.0	0.09
znf326	ENSDARG00000098348	ZNF326	ENSG00000162664	51.7	16.4	-1.7	0.0	0.16
mastl	ENSDARG00000055566	MASTL	ENSG00000120539	178.2	57.2	-1.6	0.0	0.01
ddb2	ENSDARG00000041140	DDB2	ENSG00000134574	80.2	26.5	-1.6	0.0	0.10
caska	ENSDARG00000059809	CASK	ENSG00000147044	278.2	92.2	-1.6	0.0	0.13
arl4ab	ENSDARG00000033182	-	-	178.8	59.6	-1.6	0.0	0.17
camta1b	ENSDARG00000007824	CAMTA1	ENSG00000171735	186.1	62.5	-1.6	0.0	0.02
zgc:114041	ENSDARG00000052109	-	-	464.1	157.1	-1.6	0.0	0.17
bhlhe41	ENSDARG00000041691	BHLHE41	ENSG00000123095	190.3	64.7	-1.6	0.0	0.04
exosc1	ENSDARG00000059841	EXOSC1	ENSG00000171311	101.4	34.9	-1.5	0.0	0.09
diabloa	ENSDARG00000104172	DIABLO	ENSG00000184047	3173.4	1093.7	-1.5	0.0	0.15
rxrgb	ENSDARG00000004697	RXRG	ENSG00000143171	75.1	26.0	-1.5	0.0	0.16
dedd1_1	ENSDARG00000002758	DEDD2	ENSG00000160570	670.9	233.5	-1.5	0.0	0.01
atxn1a	ENSDARG00000061687	ATXN1	ENSG00000124788	65.6	22.8	-1.5	0.0	0.16
EXOC3L4 (2 of 2)	ENSDARG00000061714	EXOC3L4	ENSG00000205436	110.4	38.7	-1.5	0.0	0.19
vegfab	ENSDARG00000034700	VEGFA	ENSG00000112715	297.9	107.8	-1.5	0.0	0.09
slc25a33	ENSDARG00000039931	SLC25A33	ENSG00000171612	447.6	163.3	-1.5	0.0	0.07
pparg	ENSDARG00000031848	PPARG	ENSG00000132170	140.2	51.2	-1.5	0.0	0.09
tbc1d10ab	ENSDARG00000060096	RP1-130H16.18	ENSG00000248751	131.4	49.1	-1.4	0.0	0.08
cipcb	ENSDARG00000078095	CIPC	ENSG00000198894	386.8	145.6	-1.4	0.0	0.04
si:dkeyp-4f2.1_2	ENSDARG00000103781	-	-	436.0	165.9	-1.4	0.0	0.16
pparaa	ENSDARG00000031777	PPARA	ENSG00000186951	120.5	47.7	-1.3	0.0	0.11
cpeb4	ENSDARG00000056691	CPEB4	ENSG00000113742	686.7	272.9	-1.3	0.0	0.03
slc16a7	ENSDARG00000021573	-	-	717.0	286.2	-1.3	0.0	0.16
parp2	ENSDARG00000079202	PARP2	ENSG00000129484	686.5	278.8	-1.3	0.0	0.14

ccdc88c	ENSDARG00000053713	CCDC88C	ENSG00000015133	373.1	154.3	-1.3	0.0	0.16
itsn1	ENSDARG00000000086	ITSN1	ENSG00000205726	377.6	156.2	-1.3	0.0	0.09
rreb1a	ENSDARG00000063701	RREB1	ENSG00000124782	137.0	57.9	-1.2	0.0	0.16
selo	ENSDARG00000041951	SELO	ENSG00000073169	184.3	78.0	-1.2	0.0	0.18
stim1b	ENSDARG00000061560	-	-	178.5	76.7	-1.2	0.0	0.15
pik3cg	ENSDARG00000017757	PIK3CG	ENSG00000105851	203.9	90.5	-1.2	0.0	0.14
kidins220a	ENSDARG00000031240	KIDINS220	ENSG00000134313	227.2	109.2	-1.1	0.0	0.19
ctsla	ENSDARG00000007836	CTSL	ENSG00000135047	347.6	766.0	1.1	0.0	0.17
JMJD8	ENSDARG00000104841	JMJD8	ENSG00000161999	128.9	284.4	1.1	0.0	0.16
ern1	ENSDARG00000013997	ERN1	ENSG00000178607	87.5	211.3	1.3	0.0	0.20
ddx18	ENSDARG00000030789	DDX18	ENSG00000088205	117.2	294.6	1.3	0.0	0.08
atp7a	ENSDARG00000003699	ATP7A	ENSG00000165240	95.8	244.3	1.4	0.0	0.17
klf13	ENSDARG00000061368	KLF13	ENSG00000169926	421.8	1117.0	1.4	0.0	0.11
znfx1	ENSDARG00000100280	ZNFX1	ENSG00000124201	57.3	153.3	1.4	0.0	0.17
mapk6	ENSDARG00000032103	MAPK6	ENSG00000069956	78.8	212.1	1.4	0.0	0.15
hs6st2	ENSDARG00000012025	HS6ST2	ENSG00000171004	20.8	56.3	1.4	0.0	0.16
cry1ba	ENSDARG00000091131	-	-	679.5	1845.6	1.4	0.0	0.01
znf277	ENSDARG00000025346	ZNF277	ENSG00000198839	29.4	81.0	1.5	0.0	0.18
ano6	ENSDARG00000061544	ANO6	ENSG00000177119	130.9	366.5	1.5	0.0	0.06
apof	ENSDARG00000090980	APOF	ENSG00000175336	35.8	104.1	1.5	0.0	0.16
ldlra	ENSDARG00000029476	LDLR	ENSG00000130164	499.1	1523.5	1.6	0.0	0.18
PLA2G4C (2 of 4)	ENSDARG00000042090	PLA2G4C	ENSG00000105499	29.6	91.3	1.6	0.0	0.04
nanp	ENSDARG00000075143	NANP	ENSG00000170191	16.5	51.0	1.6	0.0	0.16
stat2	ENSDARG00000031647	STAT2	ENSG00000170581	203.7	634.4	1.6	0.0	0.12
ssb	ENSDARG00000029252	SSB	ENSG00000138385	108.1	344.0	1.7	0.0	0.08
smarca5	ENSDARG00000052348	-	-	40.6	131.5	1.7	0.0	0.01
impdh1b	ENSDARG00000029524	IMPDH1	ENSG00000106348	123.3	400.7	1.7	0.0	0.05
eif4ebp3l	ENSDARG00000041607	EIF4EBP3	ENSG00000243056	91.4	300.2	1.7	0.0	0.17
ptges3b	ENSDARG00000089626	PTGES3	ENSG00000110958	17.7	58.1	1.7	0.0	0.07
sgk2b	ENSDARG00000006598	-	-	24.2	81.8	1.8	0.0	0.04
polr3k	ENSDARG00000037835	POLR3K	ENSG00000161980	78.0	265.2	1.8	0.0	0.01
si:ch211-260e23.9	ENSDARG00000102572	-	-	23.4	80.7	1.8	0.0	0.05

pigo	ENSDARG00000011743	PIGO	ENSG00000165282	33.1	114.1	1.8	0.0	0.09
CR855389.1	ENSDARG00000099243	-	-	39.3	135.8	1.8	0.0	0.08
slc25a44b	ENSDARG00000035905	SLC25A44	ENSG00000160785	14.6	51.0	1.8	0.0	0.06
LGALS3BP (3 of 3)	ENSDARG00000076848	LGALS3BP	ENSG00000108679	251.0	883.1	1.8	0.0	0.01
abcf2a	ENSDARG00000038785	ABCF2	ENSG00000033050	96.0	343.6	1.8	0.0	0.01
aste1	ENSDARG00000070132	ASTE1	ENSG00000034533	16.3	58.6	1.8	0.0	0.14
aqp10a	ENSDARG00000007086	AQP10	ENSG00000143595	64.5	236.5	1.9	0.0	0.01
ppargc1a	ENSDARG00000067829	PPARGC1A	ENSG00000109819	39.6	153.0	2.0	0.0	0.17
arntl2	ENSDARG00000041381	ARNTL2	ENSG00000029153	11.4	44.5	2.0	0.0	0.07
hsd11b2	ENSDARG00000001975	HSD11B2	ENSG00000176387	43.1	168.5	2.0	0.0	0.06
ctu1	ENSDARG00000020986	CTU1	ENSG00000142544	19.5	76.7	2.0	0.0	0.10
ago3b	ENSDARG00000063079	AGO3	ENSG00000126070	15.6	61.7	2.0	0.0	0.08
dhrs2	ENSDARG00000021135	DHRS4L2	ENSG00000187630	55.1	218.7	2.0	0.0	0.00
hbaa1	ENSDARG00000097011	-	-	146.1	583.8	2.0	0.0	0.06
hspa4a	ENSDARG00000004754	HSPA4	ENSG00000170606	47.3	194.7	2.0	0.0	0.09
gpt2	ENSDARG00000012199	GPT2	ENSG00000166123	21.2	90.8	2.1	0.0	0.05
arpc5la	ENSDARG00000041720	ARPC5L	ENSG00000136950	5.3	23.0	2.1	0.0	0.16
mettl2a	ENSDARG00000008105	METTL2B	ENSG00000165055	7.1	30.7	2.1	0.0	0.10
rsad2	ENSDARG00000004952	RSAD2	ENSG00000134321	22.2	97.3	2.1	0.0	0.16
trub2	ENSDARG00000028379	TRUB2	ENSG00000167112	7.8	34.1	2.1	0.0	0.15
isg20	ENSDARG00000079783	AEN	ENSG00000181026	19.3	85.7	2.1	0.0	0.07
st6galnac	ENSDARG00000086292	-	-	58.3	275.6	2.2	0.0	0.02
hif1al	ENSDARG00000100826	HIF3A	ENSG00000124440	145.8	694.4	2.3	0.0	0.08
trim35-3	ENSDARG00000052255	TRIM35	ENSG00000104228	29.6	158.7	2.4	0.0	0.01
stat1b	ENSDARG00000076182	STAT1	ENSG00000115415	13.9	74.6	2.4	0.0	0.04
gig2e	ENSDARG00000075757	-	-	6.0	32.6	2.4	0.0	0.12
soat2	ENSDARG00000059824	SOAT2	ENSG00000167780	11.4	62.9	2.5	0.0	0.06
yrdc	ENSDARG00000041428	YRDC	ENSG00000196449	13.8	79.3	2.5	0.0	0.08
ypel1	ENSDARG00000035630	YPEL2	ENSG00000175155	5.1	29.6	2.5	0.0	0.13
btg3	ENSDARG00000069065	BTG3	ENSG00000154640	6.4	37.0	2.5	0.0	0.06
ppdpfa	ENSDARG00000007682	PPDPF	ENSG00000125534	631.5	3755.6	2.6	0.0	0.02
hmox1a	ENSDARG00000027529	HMOX1	ENSG00000100292	17.5	105.7	2.6	0.0	0.02

slc43a2a	ENSDARG00000036848	SLC43A2	ENSG00000167703	43.4	266.3	2.6	0.0	0.00
si:ch211-24o10.6	ENSDARG00000088251	-	-	6.3	39.8	2.7	0.0	0.13
ZC3HAV1	ENSDARG00000100660	ZC3HAV1	ENSG00000105939	4.6	29.4	2.7	0.0	0.02
eif4ebp3	ENSDARG00000054916	EIF4EBP3	ENSG00000243056	125.6	811.5	2.7	0.0	0.02
si:ch73-39o22.3	ENSDARG00000097815	-	-	5.1	33.5	2.7	0.0	0.12
mis18bp1	ENSDARG00000042563	MIS18BP1	ENSG00000129534	2.7	17.7	2.7	0.0	0.14
cyp3c2	ENSDARG00000037874	CYP3A43	ENSG00000021461	6.2	40.9	2.7	0.0	0.03
si:dkey-33i11.1	ENSDARG00000053027	-	-	9.4	62.2	2.7	0.0	0.02
rorcb	ENSDARG00000017780	-	-	40.4	274.9	2.8	0.0	0.05
pim3	ENSDARG00000055129	PIM3	ENSG00000198355	11.1	76.2	2.8	0.0	0.01
slc29a1a	ENSDARG00000101289	SLC29A1	ENSG00000112759	13.8	97.4	2.8	0.0	0.18
cry1bb	ENSDARG00000102403	CRY2	ENSG00000121671	31.4	230.1	2.9	0.0	0.00
mhc1uba	ENSDARG00000075963	FCGRT	ENSG00000104870	46.8	346.3	2.9	0.0	0.03
gemin4	ENSDARG00000041252	GEMIN4	ENSG00000179409	3.1	23.5	2.9	0.0	0.18
dnajc21	ENSDARG00000105195	DNAJC21	ENSG00000168724	7.9	61.5	3.0	0.0	0.05
unc119b	ENSDARG00000044362	UNC119	ENSG00000109103	6.3	55.3	3.1	0.0	0.00
glula	ENSDARG00000099776	GLUL	ENSG00000135821	112.8	1003.4	3.2	0.0	0.00
RNF14 (3 of 5)	ENSDARG00000078683	RNF14	ENSG00000013561	4.6	44.0	3.3	0.0	0.02
slc6a8	ENSDARG00000043646	SLC6A8	ENSG00000130821	10.8	106.0	3.3	0.0	0.02
isg15	ENSDARG00000086374	ISG15	ENSG00000187608	10.2	104.2	3.4	0.0	0.01
sult1st1	ENSDARG00000028275	-	-	3.0	33.4	3.5	0.0	0.06
zgc:92880	ENSDARG00000069734	HBD	ENSG00000223609	2.6	39.1	3.9	0.0	0.00
cdkn1a	ENSDARG00000076554	CDKN1A	ENSG00000124762	3.0	65.3	4.5	0.0	0.00
chrm2a	ENSDARG00000098612	CHRM2	ENSG00000181072	1.0	50.1	5.7	0.0	0.00
si:ch211-117i20.2	ENSDARG00000102157	-	-	0.4	23.9	6.1	0.0	0.00
nr1d4b	ENSDARG00000059370	-	-	1.3	93.9	6.1	0.0	0.00

References

1. Matthews, M. & Varga, Z. M. Anesthesia and euthanasia in zebrafish. *ILAR J.* **53**, 192-204 (2012).

2. Hasumura, T. *et al.* Green tea extract suppresses adiposity and affects the expression of lipid metabolism genes in dietinduced obese zebrafish. *Nutr Metab (Lond).* **9**, 73 (2012).
